# Both proliferation and lipogenesis of brown adipocytes contribute to postnatal brown adipose tissue growth in mice

**DOI:** 10.1038/s41598-020-77362-x

**Published:** 2020-11-23

**Authors:** Steven G. Negron, A. Gulhan Ercan-Sencicek, Jessica Freed, Madeline Walters, Zhiqiang Lin

**Affiliations:** 1 Masonic Medical Research Institute, 2150 Bleecker Street, Utica, NY 13501 USA; 2 Department of Neurosurgery, Program On Neurogenetics, Yale School of Medicine, Yale University, New Haven, CT USA

**Keywords:** Cell growth, Cell proliferation, Developmental biology

## Abstract

Brown adipose tissue (BAT) is the primary non-shivering thermogenesis organ in mammals, which plays essential roles in maintaining the body temperature of infants. Although the development of BAT during embryogenesis has been well addressed in rodents, how BAT grows after birth remains unknown. Using mouse interscapular BAT (iBAT) as an example, we studied the cellular and molecular mechanisms that regulate postnatal BAT growth. By analyzing the developmental dynamics of brown adipocytes (BAs), we found that BAs size enlargement partially accounts for iBAT growth. By investigating the BAs cell cycle activities, we confirmed the presence of proliferative BAs in the neonatal mice. Two weeks after birth, most of the BAs exit cell cycle, and the further expansion of the BAT was mainly due to lipogenesis-mediated BAs volume increase. Microscopy and fluorescence-activated cell sorting analyses suggest that most BAs are mononuclear and diploid. Based on the developmental dynamics of brown adipocytes, we propose that the murine iBAT has two different growth phases between birth and weaning: increase of BAs size and number in the first two weeks, and BAs size enlargement thereafter. In summary, our data demonstrate that both lipogenesis and proliferation of BAs contribute to postnatal iBAT growth in mice.

## Introduction

Childhood obesity is a major public health concern^1^, and its prevalence has been increasing in the United States^2^. Childhood obesity can cause severe medical and socio-emotional consequences^3^, and is associated with increased risk of adulthood obesity^4^. Nevertheless, the pathogenesis of childhood obesity is not fully understood, and therapeutic ways to reduce or prevent childhood obesity are limited.

Obesity is a result of chronic energy imbalance: energy uptake surpasses energy expenditure, and it is marked by excessive expansion of white adipose tissue^5^. In mammals, there are two types of adipose tissues: white and brown (WAT and BAT, respectively)^6^. WAT stores triglycerides in adipocytes, and BAT dissipates chemical energy as heat^7^. Based on its developmental origin, BAT is further divided into classical BAT (cBAT, form during embryo development) and white adipocyte derived BAs (beige/brite cells, form under stress conditions, such as in low temperature)^8^. Interscapular BAT (iBAT) depot is one of the major cBAT depots in small mammals^9^. In humans, iBAT is consistently present in the children under 10 years old and dissipates in adults^10^. Recently, two independent studies have shown that the BAT activity of pediatric obese patients is significantly lower than that of the lean cohorts^11,12^. Furthermore, higher brown-like BAT composition at birth is associated with less body fat gain in early life^13^. These clinical observations have highlighted the importance of BAT in the regulation of pediatric obesity.

In mice, the iBAT depot appears at embryonic day 15.5 (E15.5) and expands rapidly through the rest of the developmental stages, likely by increasing the number of BAs^14^. From birth to pubertal maturation, the iBAT weight increases around nine times^15^. The growth of BAT is essential for keeping the whole body energy homeostasis during postnatal development, because genetically ablation of BAs causes obesity as early as 16 days after birth^16^. Although the BAT is crucial for maintaining body temperature and nutritional homeostasis, the underlying mechanisms controlling postnatal iBAT growth have not been clearly addressed^17^. There is an urgent need to understand how the BAT grows after birth, which will shed light on pediatric obesity related investigations.

In this study, we investigated the growth of iBAT in postnatal mice by dissecting the developmental dynamics of the BAs. Our data indicate that the iBAT has two different growth phases between birth and weaning: the iBAT grows by increasing brown adipocytes (BAs) number and size in the first two weeks after birth (first growth phase), and expands through BAs hypertrophic growth thereafter (second growth phase). Furthermore, our data suggest that a small fraction of BAs are proliferative in the first growth phase. In summary, this study extends our understanding of postnatal iBAT growth, and suggests the presence of proliferating BAs in newborns.

## Results

### Lipogenesis of BAs contributes to iBAT growth

**Figure 1:**
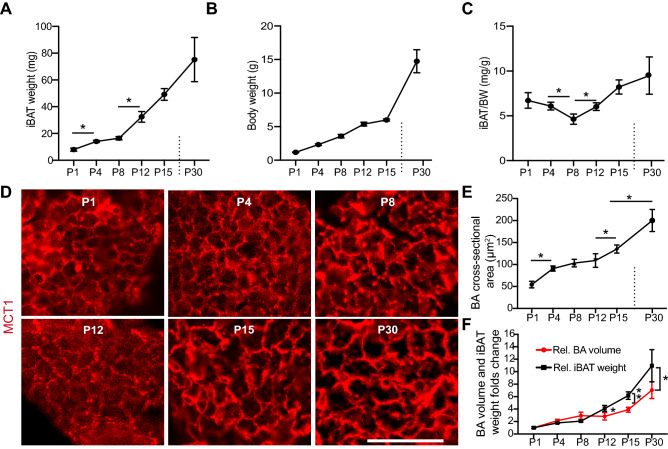
lipogenesis of BAs partially contributes to postnatal iBAT growth. Interscapular brown adipose tissues (iBAT) were collected from mice at postnatal day 1, 4, 8, 12, 15 and 30. (**A,B)** Measurement of iBAT weight (**A**) and body weight (**B**) at different ages. (**C)** iBAT weight and body weight ratio. (**D**) Immunofluorescence staining images of iBAT tissue at different ages. MCT1 (monocarboxylate transporter 1) antibody was used to visualize plasma membrane of BAs. Bar = 50 µm. € Quantification of cross-sectional area of BAs. N = 4–6. (**A**, **C** and **E**) One-way ANOVA post-hoc Tukey's multiple comparisons test, *P < 0.05; ***P < 0.001. (**F)** Folds change comparison between iBAT weight and brown adipocyte volume. iBAT weight and brown adipocytes volume at different ages were normalized to the corresponding values of P1, respectively. Student t test, *P < 0.05; **P < 0.01. N = 4–6.

In mice, the iBAT primarily grows by increasing BAs number during embryogenesis^18^. We checked the postpartum iBAT growth dynamics, which showed that the iBAT grew rapidly in the first 30 days after birth and its weight increased around 11 times from postnatal day 1 (P1) to P30 (Fig. 1A). The iBAT weight and body weight ratio decreased in the first 8 days after birth but increased between P8 and P30 (Fig. 1B,C), indicating that the cellular mechanisms controlling iBAT growth might vary at different developmental stages. Generally, the adipose tissue has two different growth mechanisms: lipogenesis growth (caused by adipocyte enlargement) and proliferation growth (due to adipocyte number increase)^19^. To understand the cellular mechanisms controlling postnatal iBAT development, we first examined the BAs size at different postnatal stages: postnatal day 1, 4, 8, 12, 15 and 30.

MCT1 (Monocarboxylate transporter 1) is a protein enriched in the BAs plasma membrane^20^. We used MCT1 antibody to visualize the cell borders of BAs^21^. From P1 to P4, the BAs cross-sectional area increased around 1.8 times (from 54.29 ± 7.447 µm^2^ to 90.66 ± 5.85 µm^2^). Compared to P4, the BAs cross-sectional area did not change significantly at P8 (103.2 ± 8.64) and P12 (109.1 ± 15.39 µm^2^). From P12 to P15 and from P15 to P30 (135.1 ± 9.24 vs 200 ± 25.02 µm^2^), the BAs cross-sectional area increased significantly in both stages (Fig. 1D,E). If hypertrophic growth of BAs was the only factor controlling iBAT expansion, the fold changes of BAs volume and iBAT weight should be similar at each growth stages. We assumed that the BAs were spherical and calculated the BAs volume fold changes with the following equation: V_n_ / V_1_ = (A_n_ / A_1_)^3/2^. V_n_ indicates the BA volume at different growth stages; V_1_ indicates the BA volume at P1. A_n_ represents the average BA cross-sectional area at different postnatal days; A_1_ represents the average BA cross-sectional area at P1. Compared to P1, the BA volume and iBAT weight had similar fold increases at P4 and P8 (Fig. 1F). At P12, the increase folds of BAs volume and iBAT weight started to diverge, with BAs volume and iBAT weight increasing 2.8 and 4 folds, respectively. The growth difference of BA volume and iBAT weight continued through P15 and P30. At P30, the BAs volume and iBAT weight were 7 and 11 times higher than that of the P1, respectively (Fig. 1F). These data indicate that lipogenesis of BAs is not the only cause of iBAT expansion, and suggest that increase of BAs number may also contribute to iBAT growth.

### Lipogenesis is associated with BAs volume enlargement

**Figure 2:**
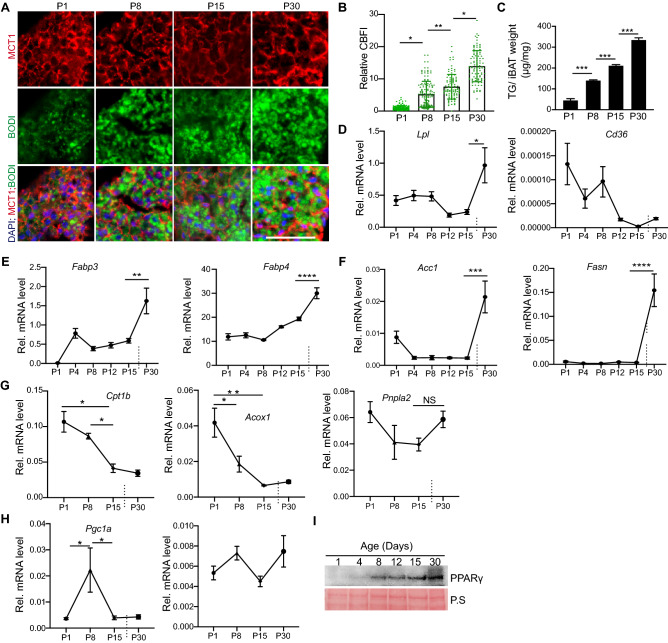
Lipogenesis is associated with BAs hypertrophic growth. (**A**) Immunofluorescence staining images of iBAT tissue at different ages. Lipid droplets were visualized with BODIPY 493/503 (BODI). Bar = 50 µm. (**B**) Quantification of cellular BODIPY fluorescence intensity (CBFI). In each group, 100–120 BAs from three independent animals were analyzed. The relative CBFI was calculated by normalizing the CBFI value of each cell against the average CBFI of P1 BAs. In the bar graph, each dot represents one single cell. Brown-Forsythe and Welch ANOVA tests with multiple comparisons test, *P < 0.05; **P < 0.01**. **(**C**) Measurement of triglycerides (TG) contents of iBAT. (**D–H**), qRT-PCR measurement of gene expression at different ages. Total RNA from iBAT was used for qRT-PCR. Gene expression level was normalized to 36B4. (C–H) One-way ANOVA with post-hoc Tukey's multiple comparisons test, *P < 0.05; ***P < 0.001, ****P < 0.0001. (**C**) n = 3 in each group. (**D**–**H**) n = 4 in each group. (**I**) Western blotting of PPARγ. Ponceau-S stained membrane was used as loading control.

In sheep, the increase of perirenal BAT weight and lipid deposition coincides between P7 and P30, but the protein content of BAT remains constant during this growth period^22^, indicating that lipogenesis is highly associated with BAT growth. We then asked the question whether lipogenesis was the main driver of BAs hypertrophic growth. BODIPY 493/503 (BODI) is a well-defined lipophilic fluorescent probe for staining neutral lipids^23^. By double staining the iBAT sections with BODI and MCT1, we were able to assess the individual BA's lipid contents: BODI staining visualized the typical multilocular lipid droplets of BAs, and MCT1 immunofluorescence signals outlined the cell borders (Fig. 2A). The density and size of the typical multilocular lipid droplets in the BAs was low at P1, increased at P8 and P15, and was highest at P30 (Fig. 2A). To quantify the BAs' lipid contents, we measured the cell BODI fluorescence intensity (CBFI). Compared to the CBFI at P1, the CBFI at P8, P15, and P30 increased 5.2, 7.5 and 14 times, respectively (Fig. 2B). We further measured the triglycerides (TG) contents of different ages iBAT. Consistent with the BODI staining results, from birth to weaning, the TG contents of iBAT increased with the age substantially (Fig. 2C). These data suggest that formation of lipid droplets is highly associated with BAs growth.

BAs increase their lipid deposition through fatty acid uptake or de novo fatty acids synthesis^24^. To understand the temporal regulation of lipogenesis, we interrogated the expression dynamics of several crucial lipogenesis genes that control lipid uptake, transport and synthesis.

Lipoprotein lipase (LPL) is a key enzyme that hydrolyses the triacylglycerol components of circulating lipoproteins^25^, and the *Lpl* gene was highly expressed in BAT^26^. Cluster of differentiation 36 (CD36) is a transmembrane protein that transports fatty acids from the extracellular environment into the cell. Inside the cell, fatty acid binding proteins (FABPs), such as FABP3^27^ and FABP4 (also known as aP2)^28^, bind and transport the free fatty acids to the mitochondria for oxidation or to the lipid droplets for storage. From P1 to P8, the expression of *Lpl* and *Cd36* did not change much; from P8 to P12, both genes decreased significantly; from P15 to P30, *Lpl* increased substantially, and *Cd36* showed out a mild increase (Fig. 2D). The expression of *Fabp3* was low at P1, increased significantly from P1 to P4, and increased substantially from P15 to P30 (Fig. 2E). The expression of *Fabp4* was constant from P1 to P15, but increased significantly from P15 to P30 (Fig. 2E). The de novo fatty acids synthesis process is oscillated by multiple key enzymes. We tested the expression of two crucial lipid synthesis genes: acetyl-CoA carboxylase 1 (*Acc1*) and fatty acid synthetase (*Fasn*)^29^. As crucial de novo fatty acids synthesis genes, both *Acc1* and *Fasn* had low expression from P1 to P15 but increased robustly from P15 to P30 (Fig. 2F). The expression dynamics of these genes suggest that the BAs primarily increase their lipid deposition through fatty acids uptake in the first two weeks after birth, and that the de novo fatty acids synthesis in BAs happens after P15.

The lipid deposition is controlled by both lipogenesis and lipolysis. Our data suggest that the postnatal iBAT development process favors lipogenesis over lipolysis. To test this notion, we measured the expression of several lipolysis related genes, including *Patatin like Phospholipase Domain Containing 2 (Pnpla2, also known as Atgl), Acyl-CoA Oxidase 1 (Acox1), and Carnitine palmitoyltransferase 1b (Cp1tb)*. *Pnpla2* encoded protein catalyzes the initial step of triglyceride hydrolysis^30^. The protein products of *Acox1* and *Cpt1b* are rate limiting enzymes of fatty acid (FA) β-oxidation, which catalyzes the first step of FA β-oxidation and facilitates the transfer of FA from cytoplasm to mitochondria, respectively^31,32^. During the postnatal iBAT growth, the expression of *Pnpla2* did not change substantially; *Acox1* and *Cpt1* decreased with age (Fig. 2G). We further examined the expression of *Pparg* and *Pgc1a,* two master regulators of adipose tissue development*.* In the four measured time points, the expression of *Pgc1a* was low at P1, robustly increased at P8, and decreased thereafter (Fig. 2H). The mRNA levels of *Pparg* did not change significantly between different ages iBAT, but the PPARγ protein level increased with age (Fig. 2H,I).

Together these data suggest that, during postnatal iBAT growth, lipid droplet deposition is the primary reason of BAs volume increase, and indicate that the lipogenesis and FA oxidation gene expression programs increase and decrease with age, respectively.

### BAs have DNA synthesis activities in the first two weeks after birth

**Figure 3:**
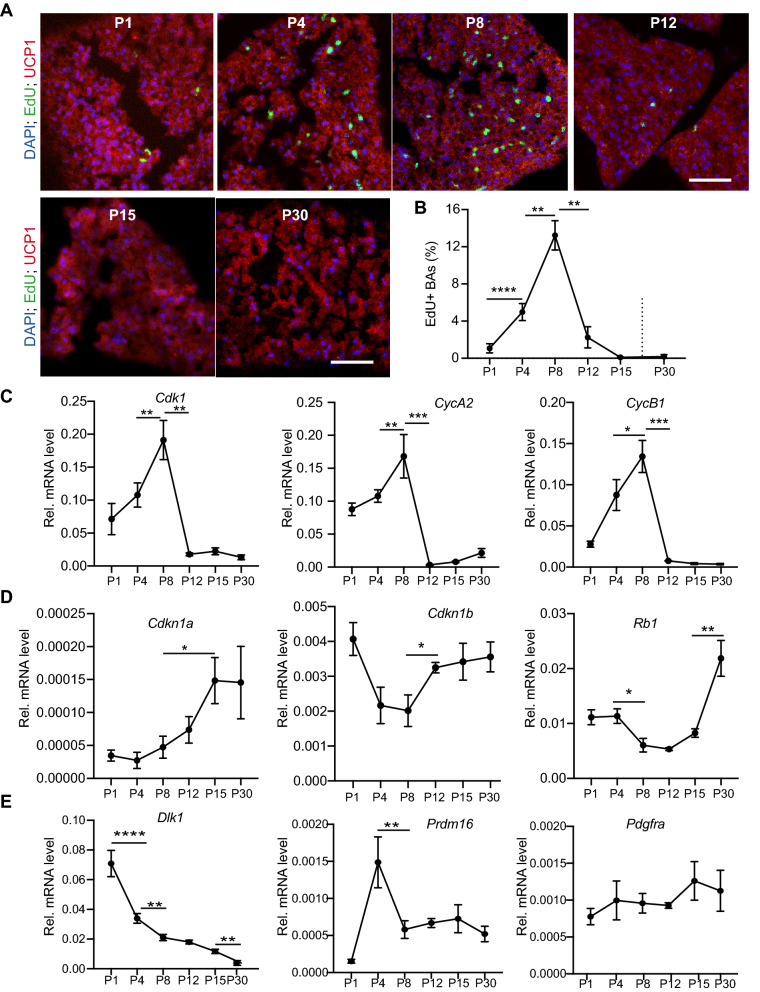
Analysis of BAs DNA synthesis activity. iBAT collected at indicated ages was used for analysis. (**A**) Representative immunofluorescence images of iBAT labeled with EdU. EdU was subcutaneously injected into mouse pups 24 h before iBAT collection. Bar = 50 µm. (**B**) Quantification of EdU positive BAs. N = 4 for each time points. (**C–E**) qRT-PCR measurement of gene expression at different ages. Total RNA from iBAT was used for qRT-PCR. (**B**–**E**) One-way ANOVA with post-hoc Tukey's multiple comparisons test. *P < 0.05, **P < 0.01, ***P < 0.001. N = 4.

Our data suggest that lipogenesis of BAs is not the only cause for iBAT expansion. We suspected that proliferation of BAs might also contribute to iBAT growth. To test this hypothesis, we analyzed the BAs' uptake rate of 5-ethynyl-2′-deoxyuridine (EdU), a nucleotide analog that labels cells passing through S phase. Six time points were included in this study: P1, P4, P8, P12, P15 and P30. Uncoupling protein 1 (UCP1) is a well-known marker of BAs. We used UCP1 antibody to label brown adipocytes. In the iBAT, EdU uptake was detected at P1, P4, P8, P12, but not at P15 and P30 (Fig. 3A). High magnification microscopy imaging showed that EdU positive cells contained both BAs (UCP1 postive) and non-BAs (UCP1 negative) (Suppl. Figure 1A). The BAs EdU uptake rate was low at P1 (1.072 ± 0.489%), increased five times at P4 (4.977 ± 0.911%), reached to a peak at P8 (13.24 ± 1.574%), and decreased to a lower level at P12 (1.57 ± 0.43%). EdU uptake was rarely detected at P15 and P30 (Fig. 2A,B). These data strongly suggest that a fraction of BAs have DNA synthesis activity in the first two weeks after birth.

To further test whether BAs have any changes in the cell cycle activity in the first two weeks after birth, we measured the expression of two sets cell cycle related genes. The first gene set includes three cell cycle genes: *Cdk1*, *CycB1* and *CycA2,* which are required for executing the cell cycle. The second gene set has three cell cycle inhibitor genes: *Rb1,* a G1/S phase check point gene^33^; *Cdkn1a and Cdkn1b,* two cyclin dependent kinase inhibitor genes^34^*.* In line with the iBAT EdU incorporation data, the expression of the three cell cycle genes peaked at P8, dramatically dropped at P12 and remained low thereafter (Fig. 3C). Although the overall expression dynamics of the three cell cycle inhibitor genes were distinct from each other, one common feature existed: they all had lower expression at P8 and higher expression at P30 (Fig. 3D). The expression patterns of these cell cycle related genes suggest the presence of proliferative BAs in the neonatal mice.

In the neonatal pups, besides originating from the preexisting BAs, the newly formed BAs could originate from two other cell populations: the adipocyte progenitor cells^35^, and dedifferentiated adipocytes^36^. If the newly formed BAs were derived from progenitor cells, we should expect a high expression of progenitor cell marker genes around one week after birth. If the BAs need to undergo dedifferentiation before they proliferate, we should detect a decrease of BAs marker gene expression at P8. *Dlk1*, *Prdm16* and *Pdgfra* are three genes highly expressed in progenitor cells^37,38^. Our data showed that the expression of *Prdm16* peaked at P4 but was relatively constant from P8 to P30 (Fig. 3E). The expression of *Dlk1* decreased with age, and the expression of *Pdgfr*a kept relatively constant from P1 to P30 (Fig. 3E). Our data showed that *Ucp1* had a peak expression at P8 (Suppl. Figure 1B), suggesting that the BAs might not undergo dedifferentiation at this age. These gene expression data suggest that the high expression of cell cycle genes in the neonatal mice is neither due to enrichment of adipocyte progenitor cells nor due to BAs dedifferentiation, and further indicate the presence of proliferative BAs.

### A subset of BAs are proliferative in the first week after birth

**Figure 4:**
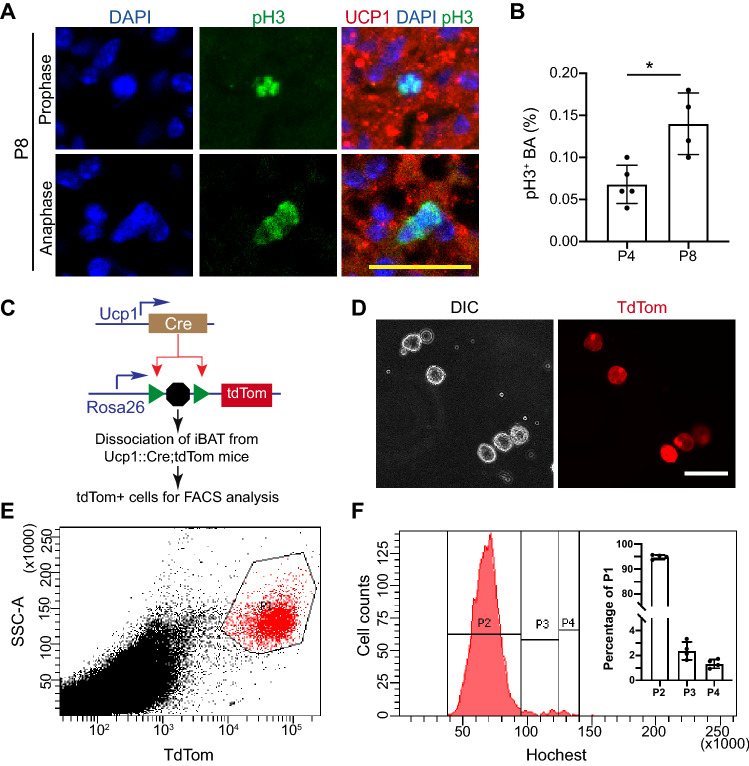
Detection of proliferating BAs in the neonatal mice. (**A)** Representative immunofluorescence images of iBAT. Bar = 25 µm. P8: postnatal day 8. pH3: phospho-Histone H3 (Ser10). (**B)** Quantification of pH3 positive brown adipocytes at postnatal day 4 and day 8. Student *t* test, *P < 0.05. Each datum point indicates one individual animal. (**C**) Experimental design for FACS analysis. In the Ucp1::Cre; Rosa 26^tdTom^ mice, BAs were genetically labeled with tdTomato (tdTom) fluorescence protein. BAs from dissociated iBAT were used for FACS analysis. (**D)** Representative images of dissociated BAs. Bar = 50 µm. (**E,F**) FACS analysis of BAs. iBAT from P8 Ucp1::Cre; Rosa 26^tdTom^ mice were dissociated and stained with Hochest. E. tdTom + BAs gating image. (**F**) DNA content histogram of gated tdTom + BAs. tdTom + BAs (Population 1, P1) were separated into three populations based on DNA contents: P2, P3, P4, representing BAs at G0/G1, S and G2/M phases, respectively. The cell number of P2, P3, and P4 was normalized to the number of gated TdTom + BAs (P1), and calculated percentages were shown in the inset bar graph. N = 4.

Our data suggest that the proliferation of existing BAs might be a reason for the proliferation growth of iBAT. Histone H3 phosphorylated on serine 10 (pH3) is a marker of M phase of the cell cycle. To further confirm the presence of proliferative BAs in neonatal mouse pups, we examined the iBAT for pH3 signals. In both P4 and P8 iBAT, pH3 positive (pH3^+^) BAs were detectable (Fig. 4A). Consistent with the EdU uptake data (Fig. 3B), P8 iBAT had more pH3^+^ BAs than P4 iBAT (Fig. 4B).

As an alternative strategy, we used fluorescence activated cell sorting (FACS) to analyze the cell cycle activity of BAs at P8. Rosa 26^tdTomato^ is a reporter mouse line, which expresses strong tdTomato (tdTom) fluorescence protein following Cre-mediated recombination^39^. Ucp1::Cre transgenic mice express Cre recombinase in the BAs^40^. We crossed the Ucp1::Cre mouse line with the Rosa 26^tdTomato^ mice to genetically label BAs with tdTom (Fig. 4C). After dissociating the P8 iBAT following a published protocol^41^, we used tdTom^+^ cells for cell cycle analysis. The presence of typical raspberry shape tdTom^+^ BAs confirmed the success of our iBAT dissociation procedure (Fig. 4D). We then ran FACS with the dissociated BAs, and gated tdTom^+^ BAs for cell cycle analysis (Fig. 4E). Published data have shown that dissociation of adipose tissue generates free lipid droplets which can be counted as FACS events^41^. During our FACS analysis, we noticed that most of the recorded events were tdTom negative (Fig. 4E). However, we were not sure how many of these tdTom negative events were cells or free lipid droplets. To focus on analyzing the brown adipocytes, we only gated for cells with high tdTom fluorescence (Fig. 4E), and then measured the DNA contents of these gated cells. Although most BAs were at G1 phase (94.83 ± 0.88%), a small fraction of BAs undergoing DNA synthesis (S phase, 2.375 ± 0.72%) and mitosis (G2/M phase, 1.325 ± 0.35%) were detected (Fig. 4F).

Together, these data suggest that a subset of BAs are proliferative in the neonatal mice.

### BAs are primarily mononuclear and diploid

Karyokinesis and cytokinesis are two related but different processes, and karyokinesis without cytokinesis may lead to polyploidy (more than two copies of DNA) or multinuclearity (more than one nucleus). Although we confirmed the presence of mitotic BAs in the neonatal mice, it was not clear whether these cells would complete the cell cycle and yield out new daughter cells. If the mitotic BAs in the neonates fail to complete karyokinesis or cytokinesis, we should be able to detect polyploidy or multinuclearity in the cell-cycle-quiescent BAs of young mice. In the iBAT of P15 or P30 mice, no more DNA synthesis activity was detected (Fig. 3A), suggesting that most of the BAs have already exited the cell cycle two weeks after birth. Therefore, we examined the BAs from P15 and P30 mice for polyploidy and nuclearity examination.

**Figure 5:**
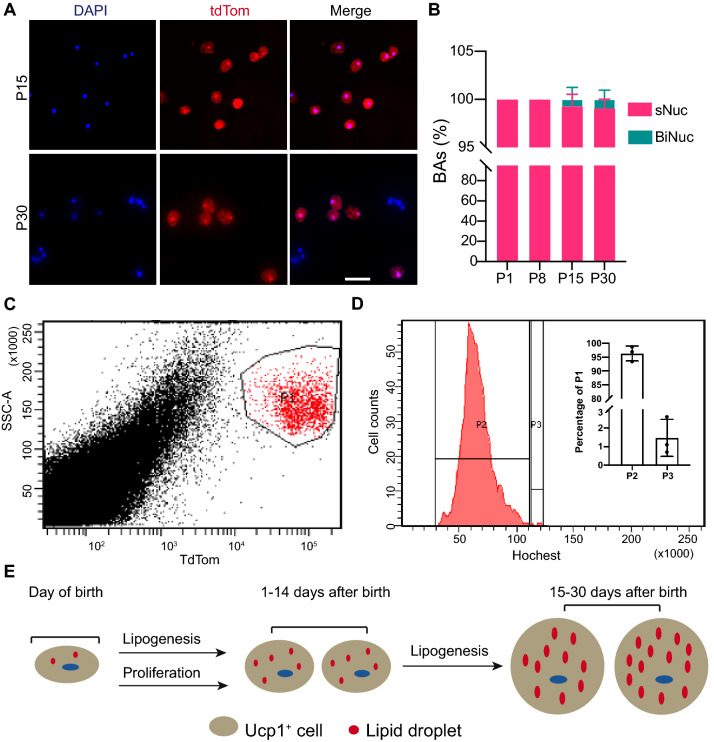
Evaluation of BAs nuclearity and DNA ploidy in juvenile mice. (**A**) Representative images of dissociated BAs. Bar = 50 µm. (**B**) Measurement of BAs nuclearity at P1, P8, P15 and P30. Dissociated BAs were stained with DAPI and counted for nucleus number. Data were collected from three independent assays. sNuc: single nucleus. BiNuc: binucleus. (**C**) TdTom + BAs gating image**. **(**D**) DNA content histogram of gated tdTom + BAs. Population 2 (P2) and Population 3 (P3) represent diploid and tetraploid BAs, respectively. The cell number of P2 and P3 was normalized to the number of gated tdTom + BAs (P1), and calculated percentages were shown in the inset bar graph. N = 4. (**E**) Schematic summary of the current findings. In the first two weeks, BAT expands by increasing BAs number (proliferation) and size (lipogenesis); from P15 to P30, lipogenesis but not proliferation of the BAs contributes to BAT expansion.

Dissociated BAs from Ucp1::Cre;Rosa 26^tdTomato^ mice were used for the following studies. We first used microscopy method to examine the BAs nuclearity. At P15, 3 out of 550 tdTom^+^ cells were binuclear, which accounted for 0.55% of the examined tdTom^+^ cells (Fig. 5A, 5B). At P30, we detected 3 binuclear BAs out of 331 tdTom^+^ cells (Fig. 5A, B, Suppl. Figure 2B). However, in 153 and P1 and 251 P8 brown adipocytes, we did not detect binuclear cells (Fig. 5B, Suppl. Figure 2A). We further checked the ploidy of the isolated tdTom^+^ cells with FACS. In the gated P30 tdTom^+^ cells (Fig. 5C), 96 ± 2.7% were diploid and 1.47 ± 1% were tetraploid (Fig. 5D), respectively. These data demonstrate that the majority of BAs from mouse iBAT contain a single diploid nucleus.

Together with the EdU incorporation data, these results further indicate that some BAs are proliferative in the first two weeks after birth, and suggest that DNA synthesis can be used as a marker for testing BAs proliferation.

## Discussion

In this study, using neonatal and juvenile mice, we interrogated the cellular and molecular mechanisms that control iBAT growth. Our data demonstrate that both lipogenesis and proliferation of BAs account for iBAT growth in the first two weeks after birth, and that lipogenesis but not proliferation of BAs contributes to iBAT expansion between two weeks after birth and weaning (summarized in Fig. 5E).

Published data showed that impaired cBAT growth had a negative effect on adult energy homeostasis^16,42^, suggesting that increasing the mass of cBAT may be an effective way to prevent obesity. Therefore, it is essential to delineate the cellular mechanisms controlling cBAT growth. As a basic question, it is not clear how the mass of cBAT changes after birth. Theoretically, the cBAT expands depends on the increase of brown adipocyte number and volume. The number of brown adipocytes may increase through two different mechanisms: proliferation of the already exist brown adipocytes and differentiation of the adipocyte progenitor cells.

In this study, by interrogating the iBAT growth in the neonatal mice, we expanded the perceptiveness of cBAT development. Our data suggest that both proliferation and lipogenesis of BAs contribute to postnatal iBAT growth, and that lipogenesis mainly accounts for BA hypertrophic growth. Based on our new data, we propose the presence of two different iBAT growth phases in early life rodents: 1) naive growth phase, and 2) maturation phase. In the naive growth phase, increase of BAs number and size coincides; in the maturation phase, lipogenesis-driven BAs hypertrophic growth contributes to the iBAT mass expansion (Fig. 5E). Our data support the notion that a fraction of embryo-derived BAs are proliferative in the iBAT naive growth phase, which at least partially accounts for the postnatal growth of iBAT. However, lineage tracing data are required to quantify how many newly formed BAs are derived from existing BAs.

Based on the published data, two distinct opinions exist regarding to the proliferation capability of terminal differentiated BAs. One category of data suggests that terminal differentiated BAs do not proliferate, and that the newly formed BAs in the adult iBAT mostly originate from PDGFRα + progenitor cells^35^. The other category of data supports the view that differentiated BAs are proliferative. For example, overexpression of a cell cycle inhibitor p27 (encoded by *Cdkn1b*) in adipocytes largely reduces the iBAT depot size without affecting BAs size^43^. The controversy of these two tires observations can now be reconciled with our new hypothesis: the overexpression of p27 suppresses the proliferation of BAs in the naive growth stage, which results in the paucity of iBAT in the adult mice. In the adult mice, embryo-derived BAs are no longer proliferative and newly formed BAs are exclusively derived from activated adipocyte progenitor cells.

Lipogenesis and lipolysis are two biochemistry processes determining the amount of lipid droplets in adipocytes^44^. Our data show that BA lipogenesis happens throughout the two postnatal growth phases, which is evidenced by the increase of multilocular lipid droplets in each individual BAs and the increase of TG levels in the iBAT. Nevertheless, the underlying mechanism may be different. Compared with the genes at P30, the expression levels of lipid synthesis genes (such as *Acc1* and *Fasn*) are constant and much lower in the naive growth phase (Fig. 2). Meanwhile, the expression of *Cd36*, a crucial fatty acid transporter gene, is much higher in the naive growth phase than in the mature phase. These gene expression data suggest that the brown adipocytes lipogenesis in naive growth phase mainly depends on extracellular lipid uptake, and that de novo lipid synthesis becomes the primary lipogenesis mechanism in maturation phase. This hypothesis is consistent with the fact that the pups mainly consume milk in the first two weeks, which contains much more crude fat than the regular chow diet^45^. Together, these data suggest that increased lipogenesis is the main reason of brown adipocytes volume increase.

Precocial species are born with a mature hypothalamic–pituitary–adrenal (HPA) axis after a long gestation period, and therefore are able to rapidly turn on non-shivering thermogenesis after birth^7^. Altricial species are born with immature HPA axis after a short gestation period, and their pups mainly maintain body temperature by huddling together instead of switching on non-shivering thermogenesis. Large animals such as sheep and humans are precocial, and rodents such as rats and mice are altricial^17^. In humans, the cBAT depots are abundant in children under 10 years old^10^, and higher brown-like BAT composition in infants is associated with lower body fat gain in early life^13^. The current study shows that the proliferation of BAs happens in the first two weeks after birth in mice, which corresponds to the first one and a half years in human^46^. Our observation raised the possibility that a cBAT naive growth phase might exist in human infants, and that this naive growth process might be essential for preparing fully functional BAT for childhood. It is also possible that precocial and altricial species share different BAT growth mechanisms, and that proliferative naive BAs only exist in altricial neonates. To have a definite answer, future works are required to examine the BAs proliferation in the neonates of precocial species such as sheep.

One of the major findings of this study is that a fraction of brown adipocytes are proliferative in the iBAT naive growth phase. It will be interesting to decipher the molecular mechanisms that control classical brown adipocytes proliferation during this growth period, which will be insightful for developing new therapeutic strategies of generating more thermogenic brown adipocytes. Beta-adrenergic signaling is one of the pathways that regulates brown adipocyte proliferation^47,48^; however, because the HPA axis is not mature in the new born mouse pups, it is unlikely that the proliferation of classical brown adipocytes is driven by beta-adrenergic signaling in the iBAT naive growth phase. Growth hormone (GH) and Insulin growth factor (IGF) signaling pathways are essential for postnatal development^49,50^, and disruption of IGF but not GH signaling in the adipose tissue results in severe iBAT paucity^51,52^, suggesting that IGF signaling may regulate the classical brown adipocytes proliferation during the iBAT naive growth phase. Future studies are secured to assess whether manipulating the IGF signaling affects the proliferation of classical brown adipocytes.

## Material and Methods

### Mice

All animal procedures were approved by the Institutional Animal Care and Use Committee of Masonic Medical Research Institute. All experiments were performed in accordance with relevant guidelines and regulations. C57BL/6 J background UCP1::Cre^40^ and Rosa 26^tdTomato^ mouse lines^39^ were reported previously, and were purchased from the Jackson Laboratory. Otherwise mentioned, C57BL/6 J background wild type mice were used throughout this study. All mice were kept at room temperature ( 22 ± 1 °C ) with a 12 h light/dark cycle. Pups were weaned at 28 days of age.

### Histology and immunostaining

Interscapular brown adipose tissue (iBAT) was fixed in 4% PFA. iBAT was cryoprotected with 30% sucrose and embedded in OCT. 10 μm sections were used for immunofluorescence staining. Antibody sources were listed in Supplementary Table 1. EdU was administered subcutaneously at 5 µg/gram body weight 24 h before iBAT collection. EdU was detected with Click-iT chemistry (Invitrogen). After primary and secondary antibody staining, sections were treated with Sudan Black B (0.5% in 70% ethanol) for 5 min to reduce background autofluorescence^53^. Imaging was performed on a Zeiss Confocal LSM 700.

### Cell size and fluorescence intensity measurements

The BAs's borders were visualized with MCT1 antibody, and the BAs' sizes were measured with Fiji software. For measuring BA's lipid contents, iBAT sections were double stained with MCT1 antibody and BODIPY 493/503. The cellular BODIPY fluorescence intensity (CBFI) was quantified based on an online protocol (https://theolb.readthedocs.io/en/latest/imaging/measuring-cell-fluorescence-using-imagej.html)*.*

### BAs isolation

BAs isolations were carried out following previous published protocol^41^ with minor modifications. Briefly, iBAT from P15 or P30 mouse pups was digested in a 20 ml flask, which was equipped with a short metal bar and placed on a magnetic stirrer. The digestion was carried out in a 36 °C incubator. The digestion buffer contained 3.5 mg/ml Dispase II, 10 mM CaCl_2_ and 1 mg/ml Collagenase II in PBS solution. 12 min after digestion, the cell suspension was transferred into a 50 ml falcon tube and filtered through a 70 µm strainer. Fresh digestion solution was added into the flask for two more round digestions. Filtered cells were incubated in PBS containing 3% BSA and 2 mM EDTA. The floated cells were fixed with 2% PFA at room temperature for 15 min. Adipocytes were subsequently stained for microscopy or FACS analysis with Hoechst to visualize nuclei.

### Measurement of iBAT triglycerides (TGs)

Lipid from iBAT of different ages mice were extracted following a published protocol^54^. Briefly, iBAT was suspended in phosphate-buffered saline (PBS) buffer at a raito of 25 µl PBS/mg tissue, and homogenized with a glass dounce homogenizer. After centrifuge, 100 µl supernate solution was transfered into a new tube. To extract the lipid, 300 µl chloroform and 150 µl methanol was added into the supernate. The mixed solution was centrifuged to separate the aqueous phase from the organic phase (containing lipid), and the latter was transfered into a new ependorf tube for air dry. The TG concentration was determined with L-type triglyceride M Assay kit (Wako diagnostic), and normalized to the iBAT weight.

### FACS analysis

Fixed and Hoechst stained BAs were filtered through 40-μm cell strainers. BAs not labeled with tdTom reporter and Hoechst were used as negative control to establish the flow cytometer settings. FACS was performed on FACSAria III (BD). The flow cytometric data were analyzed with DIVA software (BD).

### Gene expression

Total RNA was isolated using the Trizol reagent. For quantitative reverse transcription PCR (qRT-PCR), RNA was reverse transcribed (Superscript III) and specific transcripts were measured using SYBR Green chemistry and normalized to 36B4. Primer sequences were provided in Supplementary Table 2.

### Statistics analysis

Values were expressed as mean ± SEM. Student’s t-test or ANOVA with Tukey’s honestly significant difference post-hoc test was used to test for statistical significance involving two or more than two groups, respectively.

## Supplementary information

Supplementary information.
