# Supplementary material for: Both proliferation and lipogenesis of brown adipocytes contribute to postnatal brown adipose tissue growth in mice
Source: Sci Rep. 2020 Nov 23;10:20335. doi: 10.1038/s41598-020-77362-x (PMC7683731; doi:10.1038/s41598-020-77362-x)

Both proliferation and lipogenesis of brown adipocytes contribute to postnatal brown adipose tissue growth in mice

Short title: Brown adipose tissue development in early life mice

Steven G. Negron[1], A. Gulhan Ercan-Sencicek[1, 2], Jessica Freed[1], Madeline Walters[1],

and Zhiqiang Lin[1, *]

1. Masonic Medical Research Institute, 2150 Bleecker Street, Utica, NY 13501

2. Department of Neurosurgery, Program on Neurogenetics, Yale School of Medicine, Yale University, New Haven, CT, USA

*To whom correspondence should be addressed, email: zlin@mmri.edu

Supplementary information

Suppl. Table 1. Antibody list

**Primary antibody**	**Antigen**	**Origin**	**Company**	**Catalog**	**Usage (dilution)**
	MCT1	Chicken	Milipore	AB1286-I	IF (1:200)
	UCP1-PE	Mouse	R&D	IC6158P	FACS (1:100)
	Phospho Histone 3	Rabbit	Milipore	06-570	IF (1:150)
	PPARγ	Rabbit	LSBio	Ls-C368478	Western blot (1:1000)
**Seconday antibody**					
	Donkey antigoat Chicken 647	Donkey	Jackson lab	703-606-155	1:500 for IF
	Donkey Anti-Rabbit Alexa 488	Donkey	Life technology	A11055	1:500 for IF
	Donkey Anti-Rabbit HRP	Donkey	Life technology	A16035	1:10000 For WB

Suppl. Table 2. qRT-PCR Primers

**Gene name**	**Species**	**Forward**	**Reverse**
36b4	Mouse	TGCTGAACATCTCCCCCTTCTC	TCTCCACAGACAATGCCAGGAC
Cyclin B1	Mouse	AAGGTGCCTGTGTGTGAACC	GTCAGCCCCATCATCTGCG
Cyclin A2	Mouse	GCCTTCACCATTCATGTGGAT	TTGCTCCGGGTAAAGAGACAG
Cdk1	Mouse	TTTCGGCCTTGCCAGAGCGTT	GTGGAGTAGCGAGCCGAGCC
Cdkn1a	Mouse	ATGGAGACAGAGACCCCAGATAAT	CGGGACCGAAGAGACAACGG
Cdkn1b	Mouse	TAATTGGGTCTCAGGCAAACTC	CTTTTGTTTTGCGAAGAAGAATc
Rb1	Mouse	TACACTCTGTGCACGCCTTC	GGCAGTGACGATGATTTTGAACT
Ucp1	Mouse	ACTGCCACACCTCCAGTCATT	CTTTGCCTCACTCAGGATTGG
Cd36	Mouse	AAGCTATTGCGACATGATT	GATCCGAACACAGCGTAGAT
Dlk1	Mouse	AAGTGTGTAACTGCCCCTGG	ACGCAAGTTCCATTGTTGGC
Prdm16	Mouse	GGCTCAAGGAGGAGGAGAGA	AGGTCCGGGTCAGGTTCATA
Pdgfra	Mouse	CTCAGCTGTCTCCTCACAgG	CAACGCATCTCAGAGAAAAGG
Lpl	Mouse	GGGAGTTTGGCTCCAGAGTTT	TGTGTCTTCAGGGGTCCTTAG
Fabp3	Mouse	agtcactggtgacgctggacg	aggcagcatggtgctgagctg
Fabp4	Mouse	GCAGACGACAGGAAGGTGAA	TCCTTTGGCTCATGCCCTTT
Acc1	Mouse	GCCTCTTCCTGACAAACGAG	TGACTGCCGAAACATCTCTG
Fasn	Mouse	CTGACTCGGCTACTGACACG	TGAGCTGGGTTAGGGTAGGA
Pparg	Mouse	AGTGGAGACCGCCCAGGCTT	TGGAGCACCTTGGCGAACAGC
Pgc1a	Mouse	ACCGCAGTCGCAACATGCTC	GGCCTGCAGTTCCAGAGAGTTCC
Cpt1b	Mouse	TTCAACACTACACGCATCCC	GCCCTCATAGAGCCAGACC
Acox1	Mouse	CAGGAAGAGCAAGGAAGTGG	CCTTTCTGGCTGATCCCATA
Pnpla2	Mouse	CAACGCCACTCACATCTACGG	GGACACCTCAATAATGTTGGCAC

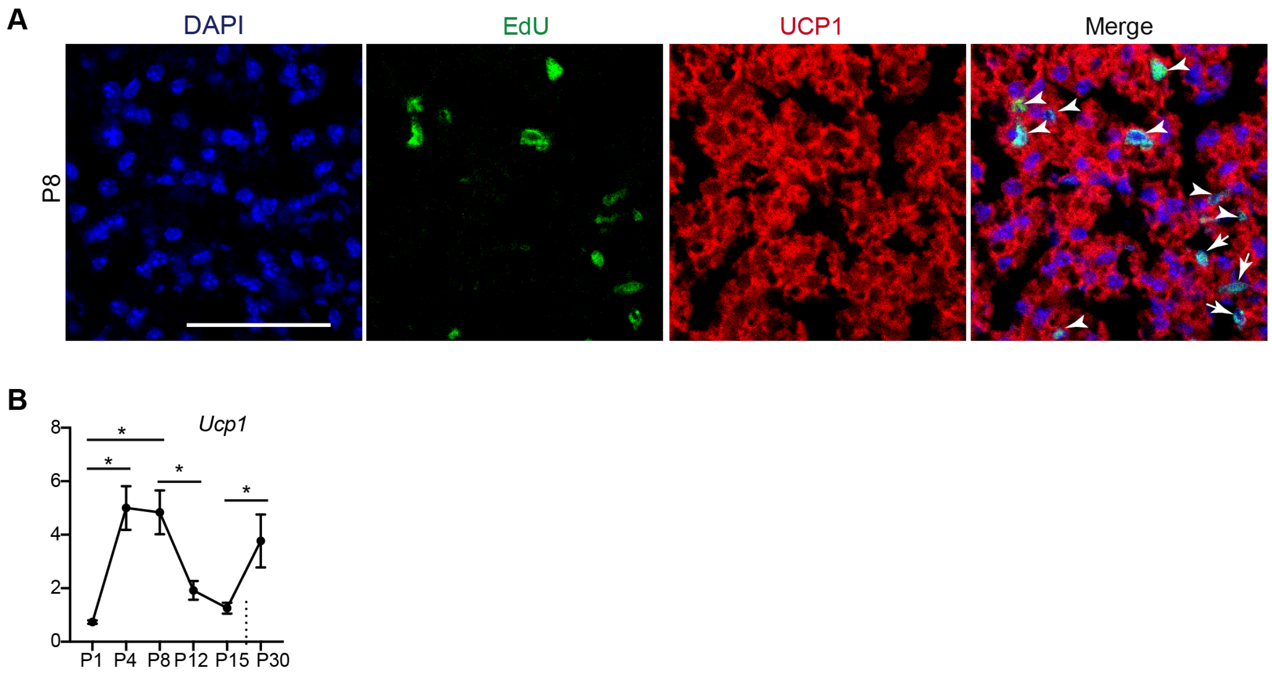

Supplementary Figure 1. Related to Figure 3

A. Representative immunofluorescence images of iBAT labeled with EdU at different ages. Arrow heads indicate EdU and UCP1 double positive brown adipocytes. Arrows indicate EdU positive and UCP1 negative cells. Bar=50μm.

B. qRT-PCR measurement of *Ucp1* mRNA. One-way ANOVA with post hoc Tukey's multiple comparisons test. *, $P<0.05$. N=4.

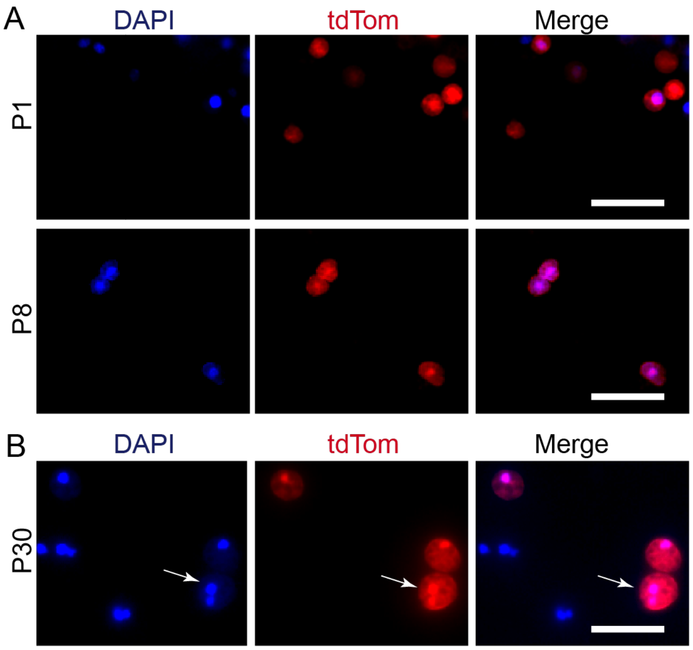

Supplementary Figure 2. Related to Figure 5
Images of dissociated BAs. Bar=50μm. **A.** Brown adipocytes from P1 and P8 mice pups. **B.** Brown adipocytes from P1 and P8 mice pups. White arrow indicate BA with double nuclei.

Original images of Fig. 2I

PPARγ western blot

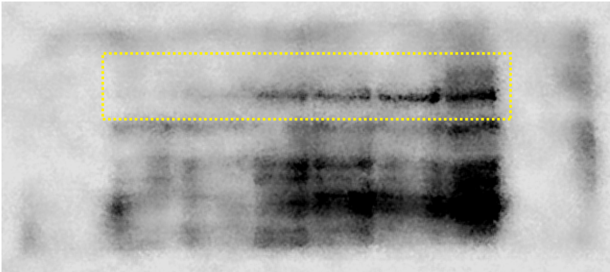

Ponceau.S staining

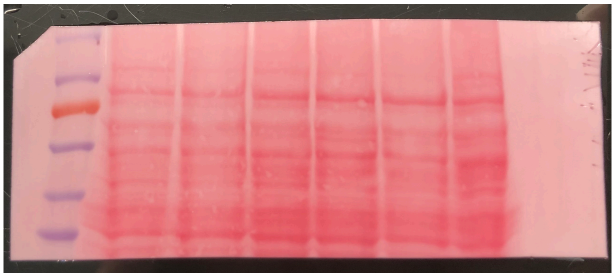

Supplement: Supplementary file 1 — Supplementary information. [file 41598_2020_77362_MOESM1_ESM.pdf]
